# Rabbit anti-human T-lymphocyte globulin and hematopoietic transplantation

**DOI:** 10.18632/oncotarget.20878

**Published:** 2017-09-14

**Authors:** Franco Locatelli, Pietro Merli, Alice Bertaina

**Affiliations:** Franco Locatelli: Dipartimento di Oncoematologia Pediatrica, IRCCS Ospedale “Bambino Gesù”, Roma, Italy; Università degli Studi di Pavia, Pavia, Italy

**Keywords:** rabbit anti-human T-lymphocyte globulin, graft-versus-host disease, hematopoietic stem cell transplantation, children

**Figure 1:**
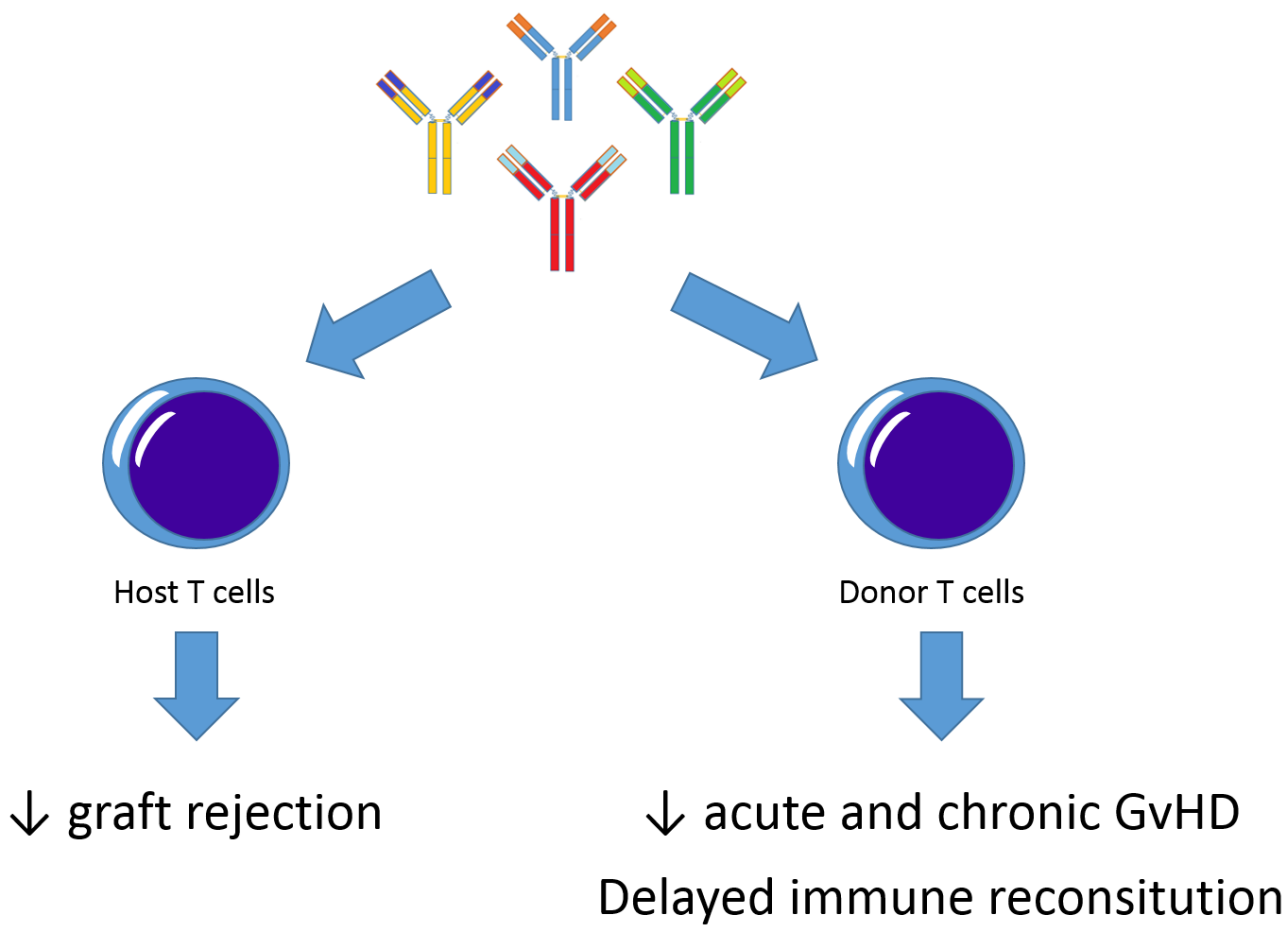
Immunomodulatory effects of ATLG on host and donor T cells

Rabbit anti-human T-lymphocyte globulin (ATLG) is used in patients undergoing allogeneic hematopoietic stem cell transplantation (HSCT) to reduce the incidence and severity of the 2 main immune-mediated complications of the procedure, namely graft rejection and acute/chronic graft-versus-host disease (GvHD) (Figure 1). In particular, there is now strong evidence, stemming from 4 randomized clinical trials, that adult patients with hematological malignancies transplanted from either an unrelated donor (UD) [1-3] or with peripheral blood stem cells (PBSC) from an HLA-identical sibling [4] benefit from pre-transplantation treatment with ATLG. The use of an UD and of PBSC are notoriously both associated with an increased risk for the recipient to develop GvHD, especially the chronic form of the disease. These 4 controlled trials, clearly showing that chronic GvHD in patients receiving HSCT can be effectively reduced through ATLG prophylaxis, with a substantial increase in the long-term quality of life, have compared either rabbit-antihuman thymocyte globulin (Thymoglobulin®, Genzyme) versus no ATLG [1, 3] or rabbit-antihuman T-cell line (Jurkat) globulin (Grafalon®, Neovii Biotech) versus no ATLG [2, 4]. None of these trials, however, had focused on a pediatric population, as well as on the identification of a dose of rabbit ATLG able to prevent GvHD, while maintaining the capacity of effectively controlling/eradicating life-threatening infections and leukemia re-growth. For addressing these issues, we decided to conduct an open-label, randomized trial comparing two different dosages of ATLG Grafalon® (30 vs 15 mg/Kg, given intravenously over 3 days, from day -4, to -2) in children (aged 0-18) with hematological malignancies receiving, after myeloablative preparation, either bone marrow- or PBSC-derived HSCT from an UD selected using high-resolution HLA typing. We found that low-dose ATLG (15 mg/Kg) can spare life-threatening infections, without significantly affecting the incidence of acute and chronic GvHD, as well as that of recurrence of the original disease [5]. This observation is further corroborated by the cumulative incidence of both EBV and adenovirus reactivation, documenting that children allocated to the low-dose arm had a reduced incidence of viral infections in comparison to those receiving ATLG at a dose of 30 mg/Kg. Preserving recovery of pathogen-specific immunity translated into a lower risk of non-relapse mortality (NRM), which, in turn, was associated with a better probability of event-free survival [5]. The composite endpoint of survival free from both chronic GvHD and relapse did not differ between children given either high- or low-dose ATLG, suggesting that a lower dose of serotherapy does not affect the quality of life of surviving patients. Overall, the results of our study, thus, provide a clear and clinically useful message, namely that children with hematological malignancies given UD-HSCT after myeloablative preparation should receive low-dose (15 mg/kg) rabbit ATLG in order to avoid the risk of increasing NRM, and, thus, of impairing the probability of event-free survival.

ATLG is usually administered, either in single or in multiple doses, on the days preceding the infusion of the graft and the observed effect may not only be dependent on the dose employed, but also on timing of infusion. Moreover, the ATLG pharmacokinetic profiles are significantly variable among patients and, at least in pediatric patients, largely influenced by recipient body weight and absolute lymphocyte count at time of treatment inception [6]. Because of the prolonged half-life of the drug, ATLG administered prior to transplantation is still detected in patients in the days after graft infusion. The systemic exposure to ATLG of donor cells, influenced by the many factors previously mentioned, has substantial effects not only on the occurrence of both acute and chronic GvHD, but also on post-allograft immune reconstitution and, thus, ultimately on survival. The mechanism of immune-suppression induced by ATLG, largely dependent on the concentration attained *in vivo*, includes T-cell depletion, modulation of adhesion and trafficking molecules, depletion and modulation of dendritic cells, as well as induction of regulatory T cells. Moreover, antigens such as CD19 or CD138 are also targeted by ATLG, this observation suggesting that the drug may also display antitumor effects in B-cell and myeloid cancers [7].

Altogether, these findings paradigmatically demonstrate the complex interplay existing between the beneficial influence of ATLG on GvHD development and the potentially harmful action on both antiviral and graft-versus-malignancy effect through depletion of donor effector T cells. Thus, although our study represents a relevant contribution to optimizing the use of polyclonal serotherapy in children with hematological malignancies transplanted from an UD selected through a sophisticated HLA-typing approach, there is still a long way to run. The different brands and doses of ATLG are not interchangeable and the results may not immediately be generalizable to other transplant settings, e.g. HSCT after reduced intensity conditioning regimen or cord blood transplantation. Moreover, in an era in which *precision medicine* is considered the future, revolutionary approach for disease treatment that takes into account individual differences in biology, additional controlled, pharmacokinetics/pharmacodynamics-based, well-designed studies are needed to further fine tune the dosing and timing of administration of this drug, extremely useful for preventing both acute and chronic GvHD, the 2 major complications of allogeneic HSCT, still associated with an increased risk of NRM and reduction in survivors’ quality of life.
